# Patient Navigation in Mothers at Risk for and Surviving with Breast/Ovarian Cancer: The Role of Children’s Ages in Program Utilization and Health Outcomes

**DOI:** 10.3390/healthcare12222317

**Published:** 2024-11-20

**Authors:** Talia Zamir, Muriel R. Statman, Marcelo M. Sleiman, Adina Fleischmann, Elana Silber, Kenneth P. Tercyak

**Affiliations:** 1 Lombardi Comprehensive Cancer Center, Georgetown University Medical Center, 2115 Wisconsin Avenue, NW, Suite 300, Washington, DC 20007, USA; 2 Sharsheret, New York, NY 10016, USA

**Keywords:** breast cancer, mothers, quality of life

## Abstract

Background/Objectives: Many women at risk for and surviving with breast/ovarian cancer are simultaneously raising children. These women often experience unique challenges due to concurrent demands as both parents and patients with cancer. Community-based cancer control organizations offer vital patient navigation (PN), including psychoeducational services. Yet, little is known about how PN addresses these mothers’ comprehensive care needs. Methods: We examined PN program data from N = 1758 women served by a national cancer organization. Results: Out of the 69% of navigated women who were mothers, most were raising adult children only (age ≥ 18; 56%); however, 31% were mothers with young children only (age < 18), and 13% were mothers with both adult and young children (χ^2^ = 341.46, *p* < 0.001). While mothers with adult children reported poorer quality of life (QoL) than mothers with young children (physically unhealthy days, *t* = −2.2, *df* = 526, *p* < 0.05; total unhealthy days, *t* = −1.2, *df* = 533, *p* < 0.05), there were no significant differences in their PN experiences. For mothers with young children, a better QoL was associated with a lower genetic risk for cancer (*r* = −0.12) and a stronger sense of psychosocial empowerment (*r* = 0.10) (all *p*’s < 0.05). In an adjusted multivariate regression model of QoL, as empowerment increased, the influence of PN quality decreased (ß = −0.007, *SE* of ß = 0.00, *p* = 0.02), suggesting that strengthening mothers of young children’s sense of agency over their breast/ovarian cancer is critical to achieving overall well-being. Conclusions: CBO-led cancer control programming that supportively cares for mothers across their cancer journey can be essential to their QoL, especially for those who are raising minors.

## 1. Introduction

The average woman in the US has a 1:8 chance of developing breast cancer (BC) and a 1:87 chance of developing ovarian cancer (OC) in her lifetime [1]. A BC or OC diagnosis interferes with all aspects of a woman’s health and well-being, including her employment, social and familial relationships, and childrearing. It is estimated that 12% of BC survivors and 5% of OC survivors live with minor children, and their mothering role while undergoing cancer treatment can be doubly burdensome [2]. Estimates of the number of patients with cancer who are raising children vary depending on location and study demographics; for example, upwards of 22% of adult cancer survivors in Germany and 25% in Japan have at least one child under 21 [3].

Mothers with cancer often face unique challenges given their dual responsibilities as mother and patient, and parenting during cancer treatment is an often-overlooked burden. Mothers with cancer may struggle to strike a balance between everyday childrearing and addressing their children’s fears and concerns about cancer [4,5,6]. Reports of mothers with cancer, both while undergoing treatment and afterward, underscore how challenging it is to navigate their own health priorities with the needs and well-being of their children. Research suggests that these mothers often reaffirm their commitment first and foremost to their children [7,8]. Additionally, survivors often bear a reduced sense of parental efficacy (i.e., a caregiver’s confidence in their ability to raise children) and experience feelings of sadness and guilt since a cancer diagnosis can disrupt how a parent feels they can meet the needs of their children [6,9].

Thus, it is incumbent on healthcare providers to consider how motherhood may influence patient decision-making, feelings of empowerment (i.e., their perceived control over their health decisions), and overall quality of life (QoL) [10,11]. Current guideline-based care practices suggest opportunities for future improvement for parents with cancer. In nursing practice, for example, the professional assessment and diagnosis associated with cancer treatment typically includes psychosocial components (e.g., fear, hopelessness, powerlessness, and readiness for enhanced knowledge) [12]. To that end, this could be extended to the psychosocial care of the family, such as the parents’ children. This may be especially important when the parent may be approaching end-of-life care to support the grief and loss that the child may experience.

Improving such practices is critical given cancer’s profound and adverse impact on the QoL of both mothers and their children. Compared to young BC survivors (those diagnosed before age 30) without children, young mothers surviving BC who have children report higher levels of fear of reoccurrence and report that BC disrupts their family dynamics to a greater extent [10,13]. Overall, young survivors report greater distress and lower QoL compared to their counterparts who do not have cancer, and they frequently report higher levels of depression, difficulty sleeping, and anxiety [14,15,16,17,18]. In particular, mothers with BC and OC may be particularly vulnerable to psychological distress when making cancer-related decisions, which may, in turn, impact their reproductive health and ability to conceive in the future [18,19]. Mothers may feel that their survivorship journey is interwoven with the functioning of their family; thus, it is important to consider how motherhood (and the ages of children) influences a survivor’s ability to maintain a high QoL.

Research suggests that the ages of mothers’ dependent children heavily impact survivors’ decision-making and psychosocial support needs. Compared to mothers who have adult children (above age 18), mothers surviving cancer who have dependent children often struggle to explain their disease to their children [20]. Many of these mothers with dependent children report seeing affective and behavioral changes in their children throughout the cancer treatment journey [21]. Many BC and OC survivors report the need for additional resources to navigate effectively discussing their disease with youth [22,23]. For example, although mothers surviving cancer who have adolescent-aged children note that they do not want conversations about cancer to dominate their relationships with their children, they seek guidance about engaging in conversations with their children about cancer in a nonthreatening way to help reduce their own uncertainty and psychological distress [24].

Community-based organizations (CBOs) have increasingly recognized and helped address the information and support needs of families affected by cancer. CBOs are integral in connecting BC and OC survivors with psychosocial and other resources, including financial support and childcare [21,25,26]. Collaboration with a CBO usually begins when a patient or family member contacts the CBO for assistance. The CBO then provides patient navigation (PN) to help connect mothers to resources and services available from within the CBO itself or allied organizations [11,27]. Research suggests that PN to these support networks offered by and through CBOs promotes the formation of strong social ties among patients with cancer, which are associated with survival advantages [28,29]. For example, when OC patients with high levels of depression and low levels of social support (high-risk) were compared to those with low depression and high levels of social support (low-risk), higher-risk patients demonstrated an upregulation in ovarian tumor progression [29]. Further, for mothers surviving or at risk for cancer, peer support networks provided by CBOs give survivors an opportunity to ask questions about treatment and share challenges, including those associated with raising children while receiving treatment [30,31]. Post-treatment support administered by and through CBOs could also be beneficial; one study found that following OC surgery, women can engage in community-based occupational therapy to reduce post-surgery distress and manage concerns [32].

These findings suggest that cancer-focused CBOs fill gaps in cancer prevention, control, and PN that are left unaddressed by the clinical services offered through hospitals and similar settings. Raising awareness about these services to the populations they aim to assist, including historically marginalized groups with low prevalence of cancer screening, may influence their overall utilization [33,34]. Mothers who belong to high-risk and vulnerable populations traditionally face systemic barriers to comprehensive cancer care in the US; CBOs’ PN can be vital in educating patients and survivors from these populations about cancer screening and help them schedule appointments and provide opportunities for psychosocial support [27,35]. The information and support offered by CBOs is particularly important to provide to mothers with adult-onset inherited cancer syndromes that may be passed from generation to generation. For example, women with Ashkenazi Jewish heritage possess unique biological risks for BC and OC due to founder mutations, making culturally competent care important for the empowerment of this population [36,37,38]. Yet, there has been little research evaluating the efficacy of CBOs in supporting mothers from diverse backgrounds who are at risk for, diagnosed with, and surviving BC and OC in this context.

In order to optimize the impact of CBO service lines, it is important to understand the overall needs and outcomes of the mothers utilizing these services, including mothers’ experience with PN and how CBOs empower mothers to make informed health decisions about BC and OC [11,39]. This study examines the utilization and care needs among a large cohort of mothers who engaged with a CBO [35]. Using secondary data analysis, we sought to examine the impact of child age on mothers’ utilization of CBO programs and their associated outcomes. The analysis consists of both a between-group comparison and within-group investigation, first by examining the outcomes of mothers according to their children’s ages and next by examining mothers with young children only to identify their specific needs.

## 2. Materials and Methods

### 2.1. Methodology

This study involves a secondary analysis of self-report surveys collected in 2022 and 2023 by a national not-for-profit CBO that provides no-cost cancer prevention and control PN services to the community. This CBO is headquartered in a northeastern state, has regional offices in four other states, maintains a toll-free telephone line, has a website, and has an active online presence to help support women across their journey with BC and OC (from risk screening and diagnosis to treatment and survivorship). Their confidential annual evaluations are administered beginning in January each year and assess women’s empowerment, satisfaction with the organization and the delivery of PN, QoL, and related outcomes. “Participation” was defined as at least one completed encounter between mothers and the CBO, including with its trained peer supporters. Encounters include PN delivered over the telephone, as well as two-way correspondences via email and text, hosted webinars, moderated social media groups, and other educational and information-oriented PN programming. Surveys were distributed by email to all community members who engaged with the organization, including women at risk for, diagnosed with, and surviving with BC and OC. Those with incomplete surveys were prompted +7, +14, and +21 days after the initial survey invitation following an established protocol [11,40]. This study was reviewed and approved by the host university’s Institutional Review Board.

### 2.2. Participants and Measures

#### 2.2.1. Sociodemographic and Clinical Characteristics

Mothers provided self-reported medical histories, including information on cancer survivorship (e.g., familial and/or genetic risks for *BRCA* mutation carriage) and survivorship status related to BC, OC, or other cancers. Demographic details, such as age, marital status, race, ethnicity, education, employment, number of children, and ages of children, were also collected [41].

#### 2.2.2. Program Service Delivery and Utilization

Program service delivery and utilization were determined by women responding to Yes/No items about whether they participated in one of six core programs or services offered by the CBO, as described below. These services are (1) Busy Box; (2) Best Face Forward; (3) Thriving Again; (4) Peer Support Network; (5) Genetics for Life; and (6) Embrace [40].

- Busy Box: Busy Box offers support for survivors facing BC while simultaneously raising young pre-teens. The program takes into account the age and gender of the children and the survivor’s expressed needs and concerns when curating a Busy Box. Participants in the program receive pamphlets related to coping with a cancer diagnosis and how to engage in family communication about this topic, including speaking with children about cancer in a parent. Age-appropriate toys and games are also provided to help occupy children of survivors undergoing treatment.
- Best Face Forward: Best Face Forward provides resources and materials addressing the cosmetic side effects of radiation and chemotherapy treatment for women with BC and OC. Informational materials about managing hair loss, changes in skin tones, and body image are included. All participants also receive a kit in the mail that includes makeup products for all skin tones and makeup application instructions. Women can also engage with the program by viewing online resources and tutorials about at-home self-care strategies.
- Thriving Again: Thriving Again is a BC and OC survivorship support program. Participants receive a survivorship kit, which is a customized booklet with advice about how to live a physically and mentally healthy life as a survivor. This includes nutritional resources (such as cookbooks and nutritious recipes), exercise regimens for women during or after their cancer treatments, and guidance for pain management, family planning, and emotional well-being.
- Peer Support Network: The Peer Support Network connects women who have been newly diagnosed with or are at high risk for developing BC or OC with one-on-one trained volunteer peer supporters who share similar diagnoses and experiences. Peer supporters connect with women over the phone or through email and offer confidential tips for coping, perspectives on healthcare providers and treatment, and friendships based on shared experiences.
- Genetics for Life: Genetics for Life addresses the concerns of women at higher risk of developing hereditary BC and/or OC. This program provides women with cancer or at risk for cancer with genetic education and information related to deleterious mutations in *BRCA* and other cancer predisposition genes. Women can engage with this program in many ways, including (1) speaking to a certified genetic counselor about family history and cancer risk (2) ordering a genetic educational booklet called *Your Jewish Genes,* which provides information about BC and OC risk in the Ashkenazi Jewish community, and (3) connecting with a peer supporter who has first-hand experience with similar concerns.
- Embrace: Embrace is designed to meet the needs of women who are living with metastatic BC or OC. The program offers one-on-one support and primarily includes a trained mental health professional who coordinates and facilitates telephone-based support group calls. Embrace participants are also connected with resources specific to women surviving advanced cancer, such as financial wellness tool kits, private Facebook groups, and information regarding clinical trials.

#### 2.2.3. Patient Navigation Quality

PN quality was measured using a study-specific seven-item scale consisting of five-point Likert ratings (1 = strongly disagree; 5 = strongly agree) to assess if the services received were (1) helpful, (2) informative, (3) timely, (4) effective, (5) supportive, (6) reliable, and (7) recommendable to others. These items were summed together to form a continuous PN quality score and then averaged, with higher scores indicating a higher quality PN experience. The internal consistency of the PN quality measure was high (Cronbach’s alpha = 0.97).

#### 2.2.4. Community-Based Organization Care Satisfaction

Satisfaction with the CBO was determined through a summary score from a four-item scale consisting of five-point Likert ratings (1 = strongly disagree; 5 = strongly agree). These scales assessed whether participants felt that the programs and services offered by the CBO were (1) valuable to them, (2) valuable to their families, (3) helpful, and (4) relevant. Together, these items assessed the CBO’s ability to understand the needs of and provide valuable support and services to women facing cancer. The measure demonstrated high reliability (Cronbach’s α) = 0.98) in assessing the extent to which women’s needs were met and felt supported by the CBO [42].

#### 2.2.5. Empowerment

The CBO assessed women’s empowerment with conceptually derived five-point Likert scales (1 = strongly disagree; 5 = strongly agree). The two empowerment items evaluated the extent to which CBO services (1) facilitated more informed choices about medical treatment and (2) increased confidence in managing health care based on the extant research literature [43]. These items were interrelated (*r* = 0.87, *p* < 0.001) and combined to form an empowerment score. A higher empowerment score was indicative of women feeling more confident in their abilities to make informed decisions for their health.

#### 2.2.6. Health-Related Quality of Life

Following guidance by the Centers for Disease Control and Prevention for assessing health-related QoL [44], women reported on their overall health (1 = poor, 5 = excellent), the total number of physically and mentally unhealthy days in the 30 days preceding the survey, and the number of days during which poor physical/mental health adversely affected their usual activities (i.e., self-care, work, recreation).

### 2.3. Data Analysis

Secondary data analyses consisted of examining between- and within-group differences and associations. Mothers were divided into the following three groups based on the age(s) of their child(ren): mothers with young children only (Group A), mothers with adult children only (Group B), and mothers with both adult and young children (Group C). Descriptive statistics were generated to describe, compare, and contrast the characteristics and prevalence of mothers from Groups A, B, and C within the CBO’s PN programming using χ^2^ tests. Next, PN program and service utilization were compared between mothers with young children only (Group A) and mothers with at least one adult child (Groups B + C). Mothers in Group A were compared to others on the dimensions of PN quality, CBO care satisfaction, empowerment, and QoL using Student’s t-test. A within-group analysis was conducted to further investigate the PN experiences and outcomes of Group A. With this analysis, we examined PN experiences and QoL and associations with demographic and clinical covariates.

## 3. Results

### 3.1. Participant Characteristics and Prevalence of Motherhood

Among all women who contacted the CBO and completed annual evaluations during the years of inquiry (N = 1758; 17% survey response rate), a majority were mothers raising one or more children (Groups A + B + C; N = 1217, 69%). As shown in Table 1, most mothers were in their early fifties, White, in partnered relationships, college-educated, and employed; 22% carried a deleterious *BRCA* mutation, and 90% were BC and/or OC survivors based on a standard definition [41]. A total of 31% (N = 377) identified as mothers with young children only (Group A; age < 18), 56% (N = 682) were mothers with adult children only (Group B; age ≥ 18), and 13% (N = 158) were mothers with both adult and young children (Group C; ages ≥ and <18). These proportions were all statistically different from one another (χ^2^ = 341.46, *df* = 2, *p* < 0.001; Group A vs. Group B, *p* < 0.001, binomial test; Group A vs. Group C, *p* < 0.001, binomial test; Group B vs. Group C, *p* < 0.001, binomial test).

### 3.2. Patient Navigation Program and Service Utilization

**Figure 1:**
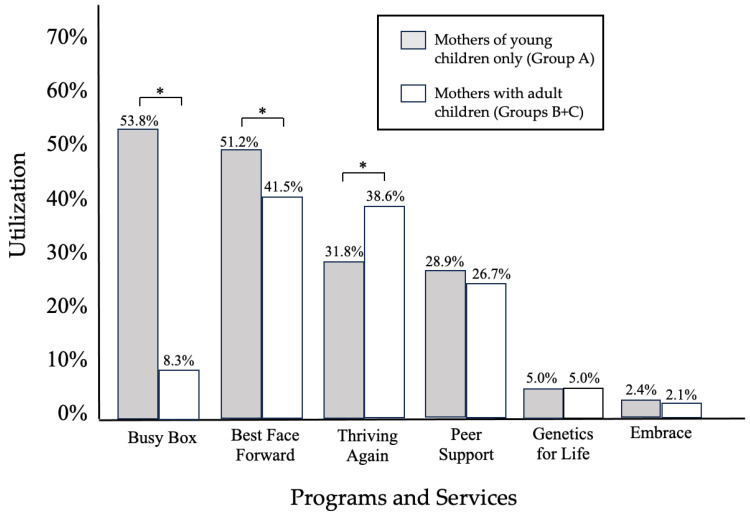
Participation in programs and services by mothers with young children only (N = 377) compared to mothers with adult children (N = 840). Note. Observations do not total 100% due to multiple responses. * Indicates significant group differences (*p* < 0.05).

Figure 1 presents a comparison of CBO program and service utilization differences between Group A and Groups B + C. The highest levels of engagement for mothers with young children (Group A) were for Busy Box (53.8%; χ^2^ = 310, *df* = 1, *p* ≤ 0.001) and Best Face Forward (51.2%; χ^2^ = 9.8, *df* = 1, *p* ≤ 0.001), and the lowest levels of engagement were for Thriving Again (31.8%; χ^2^ = 5.1, *df* = 1, *p* ≤ 0.01); 17.8% of these mothers participated in all three PN components concurrently. For all other PN programs and services, mothers’ utilization was similar based on the ages of their children.

### 3.3. Patient Navigation Outcomes and Quality of Life

Next, we compared three indices of CBO outcomes (PN quality, care satisfaction, and empowerment) and QoL between mothers in Groups A and B. Although no between-group differences in CBO outcomes were identified, differences in QoL were observed. Mothers with adult children only (Group B) experienced decreased QoL relative to mothers with young children only (Group A), both for physically unhealthy days (*t* = −2.2, *df* = 526, *p* < 0.05) and total unhealthy days (*t* = −1.7, *df* = 533, *p* < 0.05). To better understand the CBO outcomes and QoL of mothers with young children only (Group A), we then examined within-group associations among the CBO outcomes of interest and clinical characteristics with QoL.

#### 3.3.1. Patient Navigation Quality in Mothers with Young Children

The average PN quality score of Group A mothers was 4.6 out of 5 (SD = 0.78), indicating an overall positive and favorable view of the CBO’s PN efforts. A majority of these mothers strongly agreed that they found their interactions with the CBO to be helpful (73.9%) and supportive (73.8%). These mothers also agreed they would contact the CBO again in the future for themselves (73.7%) or recommend the services to their friends in need of similar assistance (78.1%). They endorsed that the PN experience connected them with valuable services and resources (75.5%) and that the information provided was given in a timely manner (74.3%). Mothers of young children also felt that PN helped them deal more effectively with their concerns (68.5%).

#### 3.3.2. Care Satisfaction and Empowerment in Mothers with Young Children

CBO care satisfaction among Group A mothers was 4.7 out of 5 (SD = 0.83), suggesting that the CBO effectively supported and provided resources for mothers and their families facing BC and OC. Many of these mothers strongly agreed that the CBO provides valuable programs (77.7%) and resources (81.1%) for survivors and their families. These mothers also strongly agreed that the CBO helped (79.5%) and understood their needs and those of their families (78.9%). Regarding mothers of young children’s sense of empowerment, this averaged 4.02 out of 5 (SD = 0.93). A substantial proportion of these mothers agreed that they felt confident in managing their healthcare (72.0%) and were well-equipped to make informed healthcare decisions regarding their cancer management (68.2%). At the bivariate level, mothers with young children who reported high satisfaction with the CBOs programs and services also reported well-developed psychosocial empowerment after engaging in these services (*r* = 0.46, *p* < 0.001).

#### 3.3.3. Quality of Life in Mothers with Young Children

The self-reported assessment of health status within the sample revealed that 20.3% of mothers with young children reported being in fair or poor health (Table 1). About 21% of these mothers reported frequent mental distress (i.e., those reporting greater than 13 days of mentally unhealthy days in the past month). On average, mothers of young children reported experiencing approximately 6.8 physically unhealthy days, 7.4 mentally unhealthy days, and 5.5 activity-limited days over the course of the past 30 days. At the bivariate level, associations were examined for mothers with young children’s clinical characteristics and PN outcome measures with QoL, as indexed by their general health. Overall, mothers in Group A who had better general health were those with higher levels of formal education (*t* = 4.3, *df* = 368, *p* < 0.001), older children (*r* = 0.13, *p* = 0.05), lower risk of cancer (*r* = −0.12, *p* < 0.05), increased quality of PN (*r* = 0.09, *p* < 0.10), and a stronger sense of psychosocial empowerment (*r* = 0.10, *p* < 0.05). In a multivariable regression model of general health among mothers with young children (Table 2), significant associations with demographic, clinical, and CBO-led cancer control outcomes were observed. After adjusting for the effects of maternal education and cancer risk, as well as child age, those with higher-quality PN experiences and a stronger sense of empowerment demonstrated improved QoL. Additionally, the interaction between PN quality and empowerment was significant, such that as empowerment increased, the positive effect of CBO-led navigation quality decreased and accounted for a substantial portion of the model’s variance in QoL (adjusted *R*^2^ = 0.92). This suggests that cancer control services in the community may have had their greatest and longest-lasting impact on mothers’ empowerment rather than the shorter-term effects of their navigation experience on overall QoL.

## 4. Discussion

This study reveals important insights regarding the efficacy of CBO-led cancer control programming to empower, support, and engage mothers who are surviving breast/ovarian cancer. It is also among the first to investigate how mothers of minors, who are particularly vulnerable due to both their cancer diagnosis and responsibilities as mothers to young children, utilize and are impacted by CBO programming. In total, nearly half (46%) of all women with children who contacted the CBO were raising youth under the age of 18 years. Although we did not observe differences in the CBO experiences of mothers based on whether they had older or younger children, we observed that mothers with younger children favored programs that assisted them in speaking to their children about their diagnosis and services that assisted with the cosmetic side effects of cancer treatment. Importantly, mothers of younger children were less likely to utilize a healthy survivorship program specific for women with breast/ovarian cancer. For all other services, there were minimal utilization differences. There could be sociocultural factors contributing to why mothers with younger children may not be utilizing genetic education services despite belonging to a population at increased risk for breast/ovarian cancer. Differences in the utilization of survivorship care planning may also suggest differential self-prioritizations among mothers with young children. These parents might opt to pursue services that support the well-being of their families and, perhaps, are less inclined to focus on their personal well-being during the survivorship phase. Understanding these differences in program use can inform how CBOs continue to meet the needs of at-risk and affected mothers depending on the ages of their children.

The findings also illustrate the downstream benefits of program utilization, including strengthened QoL. The significance of motherhood on QoL cannot be underestimated in the context of cancer, nor can the ages of their children. In our analysis comparing the CBO experiences and QoL of mothers with younger children (Group A) to those with adult children (Group B), we observed that mothers with adult children had decreased QoL across multiple measured dimensions. This may be due to age-related decrements in QoL among patients with cancer [45,46]. However, our within-group analysis among mothers with young children only (Group A) revealed that they had highly favorable experiences with the CBOs’ PN and the services they received. These findings are consistent with previous research suggesting that previvors and survivors who participate in CBO programs and PN experience psychosocial and QoL benefits [47,48]. Moreover, by analyzing the utilization of specific programs alongside quality measures, it was revealed that these mothers were well-supported in navigating their cancer journeys and felt empowered to confront their disease risk and/or breast/ovarian cancer diagnoses, treatment, and survivorship.

Understanding the effect of chronological age on mothers’ QoL is important in its own right, as well as understanding the effect of the ages of their children. That is because children of different ages will understand a cancer diagnosis differently, and this may drive differences in whether, when, and how parents openly speak with their children about the parent’s cancer, which can be beneficial [49,50]. Specifically, older children can appreciate the complexities of a cancer diagnosis and treatment more than younger children, who may need more reassurance and have greater difficulty verbally expressing their feelings. Older children, by contrast, may also have a better understanding of how cancer can affect their and their parent’s future, especially if an inherited gene change (e.g., BRCA alteration) is identified as being associated with the parent’s disease and this information is shared with the adolescent or young adult child. All children, however, may benefit from the maintenance of family routines as a way to help support the parent with cancer and the children themselves.

These findings underscore the significance of PN in mothers of young children’s feelings of empowerment and CBO care quality in relation to their QoL. In addition to the direct association of these factors with maternal QoL, their synergistic impact suggests that when these mothers’ empowerment is enhanced, the relative influence of their CBO experience is diminished. This finding suggests that PN may primarily benefit mothers’ QoL through empowerment as opposed to the short-term impact of their navigation experience. This indicates that a stronger sense of psychological empowerment over one’s cancer trajectory and survivorship can positively impact a person’s QoL, as well as their coping, decision-making, and adaptation over time, even if the disease does not remit or cannot be cured [51,52]. Moreover, behavioral interventions that are rooted in notions of elevating patient empowerment have demonstrated positive effects on QoL among patients with cancer, as well [53,54]. Thus, cancer control-focused CBOs would be well-advised to strive to provide high-quality PN services, as these services may enhance women’s feelings of personal agency over their cancer, which, ultimately, are associated with better QoL.

Such results are a testament to how PN services allow for more effective and personalized cancer care delivery since they account for an individual’s unique situations, behaviors, and preferences [55]. CBOs can play a critical role in strengthening the well-being of mothers who are at risk for and surviving breast/ovarian cancer because such services aim to connect mothers with resources and programs addressing their comprehensive care needs. Community-based cancer control organizations may thus benefit from including screening questions about parenting status, the ages and genders of children, whether the children reside in the ill parent’s household, and whether or not the ill parent is the primary caretaker. By enumerating the household in this manner, patient navigators would be more informed about the cancer parenting concerns that mothers might have. This could also be important when directing mothers to information and support resources about parenting and cancer. Tied to this would be longitudinal assessments of the needs and interests of parents with cancer and how the organization is best meeting those needs across time.

From a conceptual standpoint, Engel’s biopsychosocial model of healthcare is important to acknowledge here to place such findings in their proper context. It emphasizes the need to understand what social, psychological, and biological elements contribute to meaningfully good health. For mothers raising children while simultaneously fighting an aggressive disease, survival and QoL are of the utmost importance [56]. Lazarus and Folkman’s Coping Theory, which outlines the characteristics of problem- and emotion-focused coping, also provides valuable insights into the mechanisms by which patient empowerment and support may assist parents with their multifaceted stressors [57]. Additionally, constructs from the Transtheoretical Model’s processes of change complement these theoretical frameworks. For example, consciousness-raising is applied through public health education campaigns aimed at increasing patient awareness about BC/OC screening and providing education and feedback about programs and services to enhance QoL. Peer support programs and webinars emphasize the construct of helping relationships, attending to women’s feelings and emotions by speaking with a peer supporter on ways to resolve these feelings to help women along their cancer journey. Programs and services that facilitate maternal–child coping with cancer are meaningful and well-utilized [56,58]. Taken together, these results suggest that such offerings are an important component of CBO-led cancer control activities in communities.

### Limitations

This study has several limitations. First, the reliance on self-report data introduces the possibility of subjectivity and recall bias, which might affect the accuracy of responses. Nevertheless, patient-reported outcomes are considered the gold standard for gaining insights into individuals’ perspectives on their healthcare and their needs for information and support. Another limitation is the potential for selection bias; the data were collected from previvors and survivors who voluntarily participated in at least one of the CBO cancer control programs and completed a survey. This means that the experiences of those who opted out or did not respond to the evaluation are not represented, nor were potential differences among those who engaged with multiple programs analyzed. Although the response rate for this nonrandom survey was comparable to previous studies, it cannot ensure broad generalizability. Additionally, given that the sample was comprised of primarily White and college-educated women, this further limits the generalizability of the findings to the wider population of those confronting breast/ovarian cancer. It is also important to note that in community-wide evaluations, there often can be a polarization-response effect, whereby constituents with stronger beliefs may be more likely to provide feedback. Finally, given that this analysis primarily investigated how the ages of mothers’ children influence program utilization, future research should explore the effects of other sociodemographic and clinical characteristics on program utilization.

## 5. Conclusions

The findings from this study underscore the critical role of community-based cancer control programming in meeting the information and support needs of all mothers surviving breast/ovarian cancer through PN and tailored programming, especially among those raising young children. Future longitudinal research should further disentangle the potential effects of PN and/or empowerment on maternal QoL in the context of community-based cancer control service delivery. Continued investment in CBOs and their PN services can strengthen QoL for these mothers, addressing their dual roles as patients and caregivers. The significant participation of mothers in CBOs, particularly those with young children, indicates a clear need for ongoing enhancement of resources that aid mothers in communicating about cancer with their children. This study also highlights the essential contribution of PN in addressing health disparities, especially for populations at genetic risk for cancer, through targeted community interventions and PN. These findings can guide cancer control organizations in offering PN services and supportive care programming for affected parents with the goal of empowering them to flourish as mothers and patients alike.

## Figures and Tables

**Table 1:** Sample characteristics.

	Groups A + B + C (N = 1217)				Group A Only (N = 377)			
	M	SD	N	%	M	SD	N	%
Age	53.7	12.3			41.4	6.06		
Race								
White			1011	84.3			309	83.1
Non-White			189	15.7			63	16.9
Marital Status								
Partnered			856	70.3			297	78.8
Not partnered			361	29.7			80	21.2
Education								
<College			305	25.3			79	20.0
≥College			904	74.8			317	80.1
Employment								
Employed			767	63.0			188	49.9
Not employed or retired			450	37.0			189	50.1
*BRCA* mutation carriage and survivorship statuses								
No predisposition/No cancer diagnosis			63	5.2			14	3.7
Yes predisposition/No cancer diagnosis			60	4.9			29	7.7
No predisposition/Yes cancer diagnosis			884	72.6			258	68.4
Yes predisposition/Yes cancer diagnosis			210	17.3			76	20.2
Health-related quality of life								
General health								
Excellent/very good			267	22.2			76	20.3
Good			434	36.0			124	33.2
Fair/poor			503	41.8			174	46.5

**Table 2:** Multivariable model of general health among mothers with young children.

Independent Variables	ß	SE of ß	*p*
Education level	0.329	0.20	0.10
Child age	0.175	0.09	0.04
Cancer risk	0.132	0.11	0.22
Patient navigation quality	0.044	0.02	0.03
Empowerment	0.327	0.09	<0.001
Patient navigation quality × Empowerment	−0.007	0.00	0.02

## Data Availability

The datasets presented in this article are not readily available because they contain sensitive survey data.
